# Prediction Formulas for Individual Opioid Analgesic Requirements Based on Genetic Polymorphism Analyses

**DOI:** 10.1371/journal.pone.0116885

**Published:** 2015-01-23

**Authors:** Kaori Yoshida, Daisuke Nishizawa, Takashi Ichinomiya, Tatsuya Ichinohe, Masakazu Hayashida, Ken-ichi Fukuda, Kazutaka Ikeda

**Affiliations:** 1 Addictive Substance Project, Tokyo Metropolitan Institute of Medical Science, Tokyo, Japan; 2 Department of Dental Anesthesiology, Tokyo Dental College, Tokyo, Japan; 3 Department of Biomedical Informatics, Gifu University Graduate School of Medicine, Gifu, Japan; 4 Department of Anesthesiology and Pain Medicine, Juntendo University School of Medicine, Tokyo, Japan; Johns Hopkins Bloomberg School of Public Health, UNITED STATES

## Abstract

**Background:**

The analgesic efficacy of opioids is well known to vary widely among individuals, and various factors related to individual differences in opioid sensitivity have been identified. However, a prediction model to calculate appropriate opioid analgesic requirements has not yet been established. The present study sought to construct prediction formulas for individual opioid analgesic requirements based on genetic polymorphisms and clinical data from patients who underwent cosmetic orthognathic surgery and validate the utility of the prediction formulas in patients who underwent major open abdominal surgery.

**Methods:**

To construct the prediction formulas, we performed multiple linear regression analyses using data from subjects who underwent cosmetic orthognathic surgery. The dependent variable was 24-h postoperative or perioperative fentanyl use, and the independent variables were age, gender, height, weight, pain perception latencies (PPL), and genotype data of five single-nucleotide polymorphisms (SNPs). To examine the utility of the prediction formulas, we performed simple linear regression analyses using subjects who underwent major open abdominal surgery. Actual 24-h postoperative or perioperative analgesic use and the predicted values that were calculated using the multiple regression equations were incorporated as dependent and independent variables, respectively.

**Results:**

Multiple linear regression analyses showed that the four SNPs, PPL, and weight were retained as independent predictors of 24-h postoperative fentanyl use (*R^2^* = 0.145, *P* = 5.66 × 10^-10^) and the two SNPs and weight were retained as independent predictors of perioperative fentanyl use (*R^2^* = 0.185, *P* = 1.99 × 10^-15^). Simple linear regression analyses showed that the predicted values were retained as an independent predictor of actual 24-h postoperative analgesic use (*R^2^* = 0.033, *P* = 0.030) and perioperative analgesic use (*R^2^* = 0.100, *P* = 1.09 × 10^-4^), respectively.

**Conclusions:**

We constructed prediction formulas, and the possible utility of these prediction formulas was found in another type of surgery.

## Introduction

Opioid analgesics are widely used for the treatment of moderate to severe pain during the perioperative period. However, the analgesic efficacy of opioids is well known to vary widely among individuals [1]. For example, the minimal effective analgesic concentration of fentanyl that is required for satisfactory analgesia varies from 0.2 to 2.0 ng/ml among patients [2]. Thus, effective pain treatment is often hampered by significant differences in opioid sensitivity (S1 Fig.). Inadequate pain relief because of insufficient doses of opioids and adverse side-effects caused by unnecessarily high doses of opioids (e.g., nausea, vomiting, and constipation) are often observed in clinical settings [3]. The proper administration of opioids that meets the needs of individual patients is crucial.

Individual differences can be attributed to both environmental and genetic factors, although the relative influence of each of these factors can be variable [4]. For example, this variation in postoperative pain and analgesic consumption is reportedly affected by environmental factors including hepatic or renal function, type of surgery, and anesthesia method, demographic factors including gender, age, and ethnic origin, and preexisting psychophysical factors including anxiety and preoperative pain [4–6]. Additionally, a recent study that included identical and fraternal twins revealed that an estimated 60% of the variance in cold-pressor pain and 26% of the variance in heat pain were genetically mediated. Thus, some individual differences are likely to be attributable to genetic factors [7]. To date, various candidate genetic polymorphisms related to individual opioid sensitivity have been revealed and in particular several genetic polymorphisms within or close to regions of the *OPRM1* gene that encodes opioid receptor, mu 1, *CACNA1E* gene that encodes calcium channel, voltage-dependent, R type, alpha 1E subunit, *ADRB2* gene that encodes adrenoceptor beta 2, surface, *GIRK2* (*KCNJ6*) gene that encodes potassium inwardly-rectifying channel, subfamily J, member 6, and *CREB1* gene that encodes cyclic adenosine 3’,5’-monophosphate responsive element binding protein 1, have been reported to be strongly associated with opioid requirements in patients who underwent cosmetic orthognathic surgery with postoperative pain [8–10].

Although various factors related to individual differences in opioid sensitivity have been identified, a prediction model that calculates appropriate opioid analgesic requirements has not yet been developed. Therefore, the present study sought to construct prediction formulas for individual opioid analgesic requirements based on five genetic polymorphisms described above and clinical data using patients who underwent cosmetic orthognathic surgery. We investigated patients who underwent mandibular sagittal split ramus osteotomy, which is highly standardized at our institute regarding surgical procedures, duration of surgery, and the skill of the surgeons. Because these patients are usually young and healthy and expected to have similar pain after surgery, they may be ideal for evaluating the analgesic effects of opioids [1, 8]. We also validated the utility of the prediction formulas in another type of surgery, major open abdominal surgery.

## Materials and Methods

### Ethics statement

The study protocol was approved by the Institutional Review Boards at Tokyo Dental College (Tokyo, Japan), the Institute of Medical Science, The University of Tokyo (Tokyo, Japan), Toho University Sakura Medical Center (Sakura, Japan), and the Tokyo Institute of Psychiatry (currently Tokyo Metropolitan Institute of Medical Science, Tokyo, Japan). All of the subjects and the parents when the subjects were minors provided informed, written consent for the genetics studies.

### Subjects

Enrolled in the initial study to construct the prediction formulas for individual opioid analgesic requirements were 354 healthy patients (American Society of Anesthesiologists Physical Status I, age 15–52 years, 126 males and 228 females) who were scheduled to undergo cosmetic orthognathic surgery (mandibular sagittal split ramus osteotomy) for mandibular prognathism under general anesthesia at Tokyo Dental College Suidoubashi Hospital. Patients with chronic pain, those taking pain medication, and those who experienced Raynaud’s phenomenon were excluded. Peripheral blood samples were collected from these subjects for the gene analysis. The detailed demographic and clinical data of the subjects are provided in Table 1 and S1 Table.

**Table 1:** Demographic and clinical data of the subjects who underwent cosmetic orthognathic surgery.

Age (years)	25.9 ± 7.6 (15–52)
Male/Female	126/228
Height (cm)	164.6 ± 8.8 (143–190)
Body weight (kg)	57.7 ± 10.9 (38–128)
PPL (s)	14 [9, 23] (2–150)
*OPRM1* (rs9384179) AA/AG, GG	283/71
*CACNA1E* (rs3845446) AA/AG, GG	167/187
*ADRB2* (rs11959113) AA, AG/GG	153/201
*GIRK2* (rs2835859) TT/TC, CC	305/49
*CREB1* (rs2952768) CC/TC, TT	35/319

The data are expressed as numbers, mean ± SD (range), or median [interquartile range].

PPL, pain perception latency.

The subjects who were included in the second analysis to examine the utility of the prediction formulas that were constructed based on multiple regression analysis were different 145 patients (American Society of Anesthesiologists Physical Status I or II, age 28–80 years, 83 males and 62 females) who underwent major open abdominal surgery, mostly gastrectomy for gastric cancer and colectomy for colorectal cancer under combined general and epidural anesthesia at the Institute of Medical Science (The University of Tokyo) or Toho University Sakura Medical Center. Peripheral blood or oral mucosa samples were collected from these subjects for the gene analysis. The detailed demographic and clinical data of the subjects are provided in Table 2 and S2 Table.

**Table 2:** Demographic and clinical data of the subjects who underwent major open abdominal surgery.

Age (years)	63.8 ± 9.9 (28–80)
Male/Female	83/62
Height (cm)	158.3 ± 8.5 (133–175)
Body weight (kg)	56.3 ± 10.5 (30–80)
*OPRM1* (rs9384179) AA/AG, GG	117/28
*CACNA1E* (rs3845446) AA/AG, GG	68/77
*ADRB2* (rs11959113) AA, AG/GG	63/82
*GIRK2* (rs2835859) TT/TC, CC	132/13
*CREB1* (rs2952768) CC/TC, TT	17/128

The data are expressed as numbers and mean ± SD (range).

### General anesthesia and postoperative pain management

For the subjects who underwent cosmetic orthognathic surgery, the surgical protocol and subsequent postoperative pain management were fundamentally the same as in previous studies [8, 9]. The cold pressor-induced pain test was performed before the induction of anesthesia as previously described [11, 12]. Briefly, crushed ice cubes and cold water were blended 15 min before testing in a 1 L isolated tank, and the mixture was stirred immediately before each test to ensure a uniform distribution of temperature (0°C) within the tank. The dominant hand was immersed up to the wrist. The subjects were instructed to keep their hand calm in the ice-cold water and withdraw it as soon as they perceived any pain. The same investigator conducted the test for all of the patients. The baseline latency to pain perception, defined as the time of immersion of the hand in the ice water (pain perception latency [PPL]), was recorded. A cut-off point of 150 s was set to avoid tissue damage. After the induction of anesthesia, peripheral blood samples were collected from these subjects for the gene analysis. After emergence from anesthesia and tracheal extubation, 1.25 mg droperidol was administered intravenously to prevent nausea/vomiting, and intravenous patient-controlled analgesia (PCA) with fentanyl (1 mg fentanyl and 5 mg droperidol diluted in normal saline in a total volume of 50 ml) commenced using a CADD-Legacy PCA pump (Smiths Medical Japan, Tokyo, Japan). The PCA settings included a bolus dose of 20 μg fentanyl on demand and a lockout time of 10 min. Continuous background infusion was not used. Droperidol was coadministered with fentanyl to prevent nausea/vomiting because our preliminary study found a high incidence (up to 30%) of nausea/vomiting with PCA fentanyl in young females. Patient-controlled analgesia was continued for 24 h postoperatively. Intraoperative fentanyl use and postoperative PCA fentanyl use during the first 24-h postoperative period were recorded. The doses of fentanyl administered intraoperatively and postoperatively were normalized to body weight.

For the subjects who underwent major open abdominal surgery, the surgical protocol and subsequent postoperative pain management were fundamentally the same as in previous studies [13, 14]. Postoperative pain was managed primarily with continuous epidural analgesia with fentanyl or morphine. Fentanyl or morphine was diluted with 0.25% bupivacaine in a total volume of 100 ml and infused at a constant rate of 2 ml/h through a catheter placed in the lower thoracic or upper lumbar epidural space. Whenever the patient complained of significant postoperative pain despite continuous epidural analgesic, appropriate doses of opioids, including morphine, buprenorphine, pentazocine, and pethidine, and/or nonsteroidal antiinflammatory drugs (NSAIDs), including diclofenac and flurbiprofen, were administered as rescue analgesics at the discretion of the surgeons based on the patient’s request. The doses of rescue analgesics (opioids and/or NSAIDs) administered during the first 24-h postoperative period and dose of analgesics administered during the intraoperative period were recorded. To allow intersubject comparisons of the analgesic doses required during the intraoperative period and first 24 h postoperative period, the doses of opioids and NSAIDs administered as rescue analgesics during these periods were converted to the equivalent dose of systemic fentanyl according to previous reports [13–24]. The total dose of analgesics administered was calculated as the sum of the systemic fentanyl-equivalent doses of all of the opioids and NSAIDs administered to patients as analgesics during the intraoperative period and first 24-h postoperative period.

Peripheral blood or oral mucosa samples were collected from these subjects after surgery for the gene analysis.

### Genotyping

Genomic DNA was extracted from the oral mucosa or whole-blood samples using the Wizard Genomic DNA Purification Kit (Promega, Tokyo, Japan) or the QIAamp DNA Mini Kit (Qiagen, Tokyo, Japan) according to the manufacturer’s instructions [25].

For genotyping the five selected single-nucleotide polymorphisms (SNPs; rs9384179 in the human *OPRM1* gene, rs3845446 in the human *CACNA1E* gene, rs11959113 in the human *ADRB2* gene, rs2835859 in the human *GIRK2* [*KCNJ6*] gene, and rs2952768 around the human *CREB1* gene), direct sequencing, the TaqMan allelic discrimination assay, and Infinium assay II were used. Genotype data from whole-genome genotyping, which was performed using Infinium assay II and an iScan system (Illumina, San Diego, CA, USA), were used for the rs3845446, rs11959113, rs2835859, and rs2952768 SNPs. Direct sequencing was conducted to genotype the rs9384179 SNP as described in a previous report [8]. The TaqMan allelic discrimination assay was conducted to genotype the rs3845446, rs2835859, and rs2952768 SNPs as described in previous reports [9, 10] and the rs11959113 SNP. To perform the TaqMan assay with a LightCycler 480 (Roche Diagnostics, Basel, Switzerland), we used TaqMan SNP Genotyping Assays (Life Technologies, Carlsbad, CA, USA) that contained sequence-specific forward and reverse primers to amplify the polymorphic sequence and two probes labeled with VIC and FAM dye to detect both alleles of the rs3845446, rs11959113, rs2835859, and rs2952768 SNPs (Assay ID: C___7539287_30, C__27108051_10, C__16076710_10, and C__11510543_20, respectively). Real-time polymerase chain reaction was performed in a final volume of 10 μl that contained 2× LightCycler 480 Probes Master (Roche Diagnostics), 40× TaqMan SNP Genotyping Assays, 5–50 ng genomic DNA as the template, and H_2_O (Roche Diagnostics). The thermal conditions were the following: 95°C for 10 min, followed by 45 cycles of 95°C for 10 s and 60°C for 60 s, with final cooling at 50°C for 30 s. Afterward, endpoint fluorescence was measured for each sample well, and each genotype was determined based on the presence or absence of each type of fluorescence. The details of Infinium assay II for whole-genome genotyping were provided in a previous report [9].

### Statistical analysis

Three hundred fifty-four subjects who underwent painful cosmetic surgery were used for the initial analysis to construct prediction formulas for individual opioid sensitivity. As an index of opioid sensitivity, postoperative PCA fentanyl use during the first 24-h postoperative period was used because analgesic requirements likely reflect the efficacy of fentanyl in each individual patient. Prior to the analyses, the quantitative values of PPL (in seconds) and postoperative fentanyl requirements (μg/kg) were natural-log-transformed for approximation to the normal distribution according to the following formulas: *Value for analyses = Ln (1 + endpoint PPL value [s])* and *= Ln (1 + postoperative fentanyl requirement [μg/kg])*, respectively. To predict 24-h postoperative fentanyl requirements from the clinical or genomic parameters that may affect the analgesic efficacy of fentanyl (i.e., age, gender, height, weight, PPL, and genotype of the five SNPs), multiple linear regression analysis using the stepwise method of bidirectional selection was performed. The cut off *P* value for inclusion was set at 0.05. Moreover, to confirm the utility of including the five SNPs in the predictive models of 24-h postoperative and perioperative fentanyl use, we examined whether adding the five SNPs to other patient characteristics in the models improves its predictive ability using forced entry method. The dependent variable was 24-h postoperative fentanyl use, and the independent variables were age, gender, height, weight, pain perception latencies, and genotype data of five single-nucleotide polymorphisms (SNPs). For the analysis, the gender and genotype data for each SNP were used as dummy variables based on the previous analyses (S3 and S4 Tables) by replacing females and the genotypes associated with greater fentanyl use with 1, and males and the genotypes associated with lesser fentanyl use with 0. Therefore, we replaced homozygous carriers of the A allele of the rs9384179 SNP with 1 and non-carriers with 0, homozygous carriers of the A allele of the rs3845446 SNP with 1 and non-carriers with 0, carriers of the A allele of the rs11959113 SNP with 1 and homozygous carriers of the G allele with 0, homozygous carriers of the T allele of the rs2835859 SNP with 1 and non-carriers with 0, and homozygous carriers of the C allele of the rs2952768 SNP with 1 and non-carriers with 0, respectively. Similar analysis using subjects who underwent cosmetic orthognathic surgery was also conducted for analgesic requirements during the perioperative period instead of during the postoperative period because the analgesic effect of the intermediate-acting analgesics, administered pre- and intraoperatively, could outlast the duration of surgery and thus affect postoperative analgesic use, especially in patients who received large doses of analgesics intraoperatively. Perioperative fentanyl use was calculated as the sum of the intraoperative and postoperative doses of fentanyl or fentanyl-equivalent doses of analgesics.

Second analyses were conducted using the different 145 subjects who underwent major abdominal surgery. The total dose of rescue analgesics administered during the first 24-h postoperative period was used as an index of opioid sensitivity. Prior to the analyses, the quantitative values of postoperative analgesic requirements (μg/kg) were natural-log-transformed for approximation to the normal distribution according to the following formula: *Value for analyses = Ln (1 + postoperative analgesic requirement [μg/kg])*. To explore the regression of the actual value of 24-h postoperative analgesic requirements after major abdominal surgery to the predicted value that was calculated using the multiple regression equation constructed in the initial analysis, simple linear regression analysis was performed. Actual 24-h postoperative analgesic use (μg/kg; log-transformed) and the predicted value that was calculated were incorporated as dependent and independent variables, respectively. Similar analysis using subjects who underwent major abdominal surgery was also conducted for analgesic requirements during the perioperative period instead of during the postoperative period. Perioperative fentanyl use was calculated as the sum of the intraoperative and postoperative doses of fentanyl or fentanyl-equivalent doses of analgesics.

For all of the statistical analyses described above, IBM SPSS v.20.0 for Windows software (IBM Japan, Tokyo, Japan) was used.

## Results

**Fig 1:**
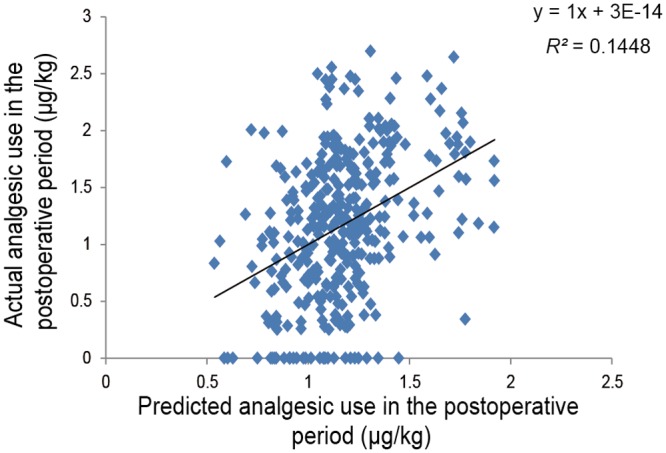
Regression analysis of 24-h postoperative fentanyl use after cosmetic orthognathic surgery. The figure shows a scatterplot for the actual value of 24-h postoperative fentanyl use (μg/kg; log-transformed) in patients who underwent cosmetic orthognathic surgery and the predicted value that was calculated. Each point represents an individual patient. The solid line in the scatterplot represents the regression line, and the mathematical formula represents the regression equation.

**Fig 2:**
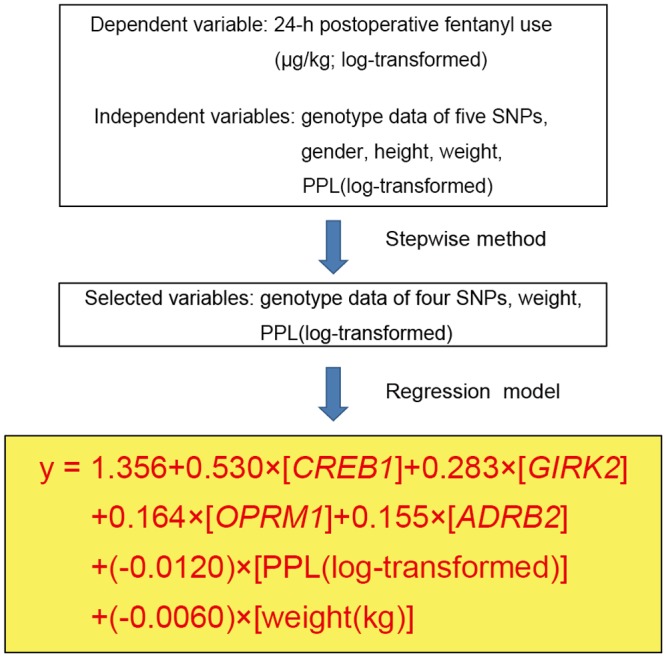
Construction of prediction formula. Multiple linear regression analysis during the postoperative period using the stepwise method was performed to construct a prediction formula for individual opioid sensitivity. “y” represents the predicted value of 24-h postoperative fentanyl requirements (μg/kg; log-transformed) that was calculated using the multiple regression equation.

**Fig 3:**
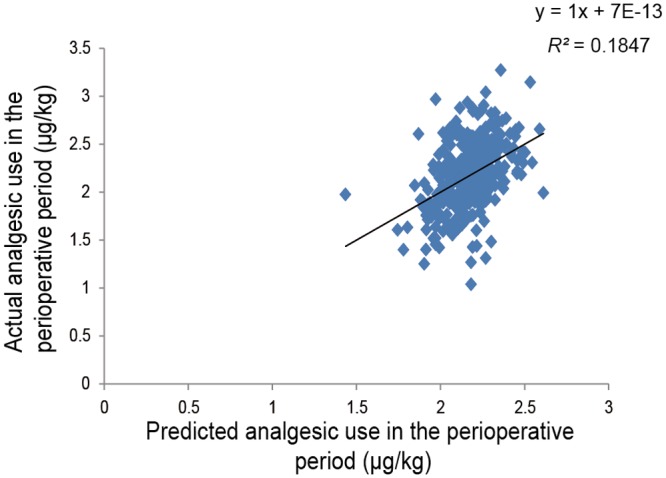
Regression analysis of perioperative fentanyl use after cosmetic orthognathic surgery. The figure shows a scatterplot for the actual value of perioperative fentanyl use (μg/kg; log-transformed) in patients who underwent cosmetic orthognathic surgery and the predicted value that was calculated. Each point represents an individual patient. The solid line in the scatterplot represents the regression line, and the mathematical formula represents the regression equation.

The *R^2^* value was improved from 0.062 to 0.169 and from 0.127 to 0.208 by adding the five SNPs to other patient characteristics in the predictive models of 24-h postoperative and perioperative fentanyl use, respectively. Initial multiple linear regression analysis of the postoperative period using the stepwise method showed that the genotype of the four SNPs i.e. rs2952768 (*P* = 1.3 × 10^-6^), rs2835859 (*P* = 0.003), rs9384179 (*P* = 0.042), and rs11959113 (*P* = 0.018), PPL (*P* = 0.016), and weight (*P* = 0.044) were retained as independent predictors of 24-h postoperative fentanyl use for cosmetic orthognathic surgery (*R^2^* = 0.145, *P* = 5.66 × 10^-10^; Fig. 1). The detailed multiple regression equation is shown in Fig. 2. Similar multiple linear regression analysis of the perioperative period considering the analgesic effect of intermediate-acting analgesics administered pre- and intraoperatively showed that the genotype of the two SNPs, rs2952768 (*P* = 9.1 × 10^-5^) and rs3845446 (*P* = 0.001), and weight (*P* = 2.8 × 10^-12^) were retained as independent predictors of perioperative fentanyl use for cosmetic orthognathic surgery (*R^2^* = 0.185, *P* = 1.99 × 10^-15^; Fig. 3). The multiple-regression equation was the following: *predicted value of perioperative fentanyl requirements (μg/kg; log-transformed) = 2.749 + 0.221 × [CREB1] + 0.109 × [CACNA1E] + (-0.011) × [weight (kg)].*

**Fig 4:**
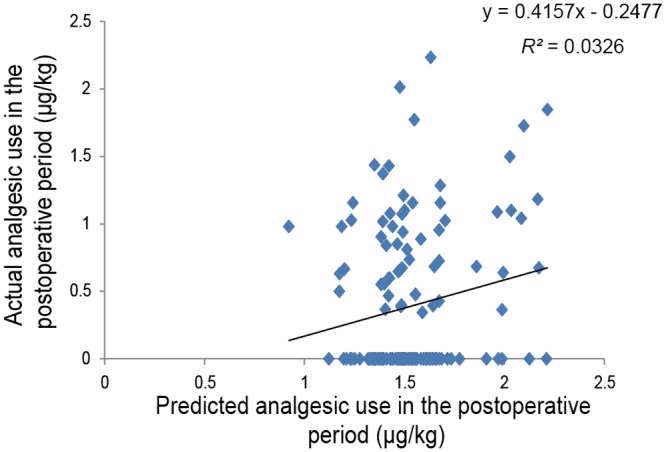
Regression analysis of 24-h postoperative analgesic use after major abdominal surgery. The figure shows a scatterplot for the actual value of 24-h postoperative analgesic use (μg/kg; log-transformed) in patients who underwent major abdominal surgery and the predicted value that was calculated. Each point represents an individual patient. The solid line in the scatterplot represents the regression line, and the mathematical formula represents the regression equation.

**Fig 5:**
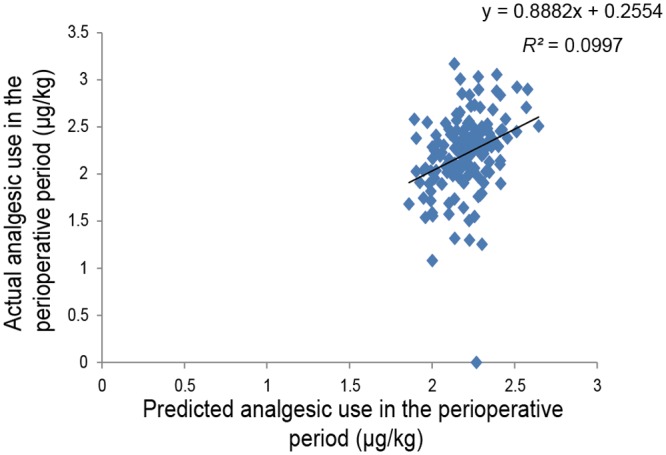
Regression analysis of perioperative analgesic use after major abdominal surgery. The figure shows a scatterplot for the actual value of perioperative analgesic use (μg/kg; log-transformed) in patients who underwent major abdominal surgery and the predicted value that was calculated. Each point represents an individual patient. The solid line in the scatterplot represents the regression line, and the mathematical formula represents the regression equation.

Second simple linear regression analysis of the postoperative period using subjects who underwent major abdominal surgery showed that the predicted value that was calculated was retained as an independent predictor of actual 24-h postoperative analgesic use for major abdominal surgery (*R^2^* = 0.033, *P* = 0.030; Fig. 4). Similar simple linear regression analysis of the perioperative period showed that the predicted value that was calculated was retained as an independent predictor of actual perioperative analgesic use for major abdominal surgery (*R^2^* = 0.100, *P* = 1.09 × 10^-4^; Fig. 5).

## Discussion

A prediction formula for individual opioid analgesic requirements during the first 24-h postoperative period (Fig. 2) was constructed using multiple linear regression analysis in healthy subjects who underwent painful cosmetic orthognathic surgery. By conducting similar analysis, we also constructed a prediction formula for the perioperative period, although the variables that were retained as independent predictors after the analysis of the perioperative period were not the same as those of the postoperative period (Fig. 3). The *R^2^* index in the prediction formula for perioperative analgesic requirements was higher than the *R^2^* index for the first 24-h postoperative analgesic requirements. Furthermore, by conducting simple linear regression analyses in subjects who underwent major open abdominal surgery, we demonstrated the possibility that the prediction formulas may be useful in another type of surgery (Figs. 4 and 5), although the predictability of the formula remains to be verified in future studies. Using the prediction formulas and the patients’ genetic polymorphisms and clinical data, better analgesia could be provided to individual patients. Based on the predicted values, less fentanyl could be administered to patients with higher opioid sensitivity, and more fentanyl could be administered to patients with lower opioid sensitivity. For example, setting the appropriate dose of analgesics for individual patients by setting an upper level has great potential for safe, personalized pain control while minimizing potential side effects.

Empirical approaches to the effective treatment of pain are currently limited because the initial drug selection and subsequent dosage titrations, drug additions, and switches in therapy are driven by only a few patient-specific clinical features [26]. Thus, pharmacogenetic studies are being developed to advance personalized medicine. Pharmacogenetics is defined as the use of pharmacogenomic or pharmacogenetic tests in conjunction with drug therapy and has the potential to change the way in which healthcare is provided by stratifying patients into likely responders, likely non-responders, and likely to experience adverse drug reactions [27]. The use of pharmacogenomic principles represents an opportunity to enhance public health and patient care. Therefore, prediction formulas can be constructed for individual analgesic requirements based on genetic polymorphisms.

Recently, methods of predicting drug requirements using genetic tests may be adopted for drugs for which dose adjustments are difficult because of large individual differences in sensitivity. For example, a dosing algorithm has been constructed for warfarin, consisting of the patient’s age, body surface area, state of amiodarone co-administration, and genotype of three SNPs (*R^2^* = 0.434) [28]. A prospective study reported that an algorithm guided by pharmacogenetic and clinical factors improved the accuracy and efficiency of warfarin dose initiation, although a reduction of out-of-range prothrombin time international normalized ratios (INRs) was not achieved [29]. Thus, prediction formulas have also been constructed and verified for medications other than opioid analgesics. Although the precision of our prediction formula for individual opioid analgesic requirements appears to be relatively low in terms of the *R^2^* index compared with the warfarin dosing algorithm, such algorithms are expected to be improved in future studies and contribute to personalized medicine.

Gene and opioid-effect relationships are difficult to determine from data that are sampled from patients with cancer pain because the mechanism, severity, and nature of cancer pain can differ substantially between patients [30]. Thus, patients with acute postoperative pain after standardized surgical procedures in the present study may be optimal subjects for investigating such relationships [1]. The precision of our prediction formulas for individual opioid analgesic requirements was relatively low in terms of the *R^2^* index for major open abdominal surgery compared with cosmetic orthognathic surgery, and several factors may have contributed to this imprecision. The first consideration is differences in the surgical procedures and the degree of invasiveness. The present study included patients with various diseases and pathologies that required major abdominal surgery. Another consideration is the analgesics employed and their routes of administration. Various analgesics and routes of administration are used in major abdominal surgery, and analgesic action, absorption, metabolism, and excretion can vary considerably among analgesics and routes of administration. For example, buprenorphine is long-acting and antagonizes other opioids, which may prevent the effects of continuous epidural opioids and other rescue opioids. A third consideration is the pathways of pain. Major abdominal surgery involves visceral pain, and some pain pathways may not be exactly the same as those associated with cosmetic orthognathic surgery. Therefore, to apply our formulas to other types of surgery, further investigation is necessary. Future studies should verify the utility of the prediction formulas in surgeries that have fewer variations with regard to patients and surgical procedures.

Additionally, studies that investigate the genes that encode opioid metabolizing enzymes and transporters are also worth performing. Because most opioid drugs are metabolized by cytochrome P450 enzymes (CYPs), including CYP2D6, glucuronidated by UDP-glucuronosyltransferases (UGTs), and transported between the blood and brain by ATP-binding cassette, sub-family B (MDR/TAD), member 1 (ABCB1), the genes that encode these metabolic enzymes and transporters are worth examining with regard to differences in analgesic requirements [2, 31, 32]. The onset of negative effects (e.g., nausea, vomiting, and constipation) may also be a useful and interesting outcome because such effects can cause some patients to stop requesting analgesics despite not actually achieving full analgesia. However, we did not construct prediction formulas for negative effects because the number of patients with adverse side-effects was small in the present study. Additionally, other variables, such as cigarette smoking status and pre-existing opioid tolerance, can influence opioid analgesic requirements. We found that some SNPs were associated with self-reported pain level (data not shown), and pain level in addition to analgesic requirements may be predicted by gene analyses.

Moreover, gene-gene interactions and gene-environment interactions can also affect opioid sensitivity, although the present study assumed that each factor was independent. Thus, other prediction formulas should be constructed by taking these factors into account. Such considerations should aid in the development of more effective personalized pain treatment for patients who suffer from cancer pain or postoperative pain after various surgeries by predicting opioid sensitivity based on simple genetic tests.

In conclusion, by conducting multiple linear regression analyses of healthy subjects who underwent painful cosmetic orthognathic surgery, we constructed prediction formulas for individual opioid analgesic requirements during the first 24-h postoperative period and perioperative period. Furthermore, by conducting simple linear regression analyses in subjects who underwent major open abdominal surgery, we found that these prediction formulas may be useful for other types of surgery. Although further validation is needed, our data provide valuable information for the individualization of appropriate fentanyl doses to achieve adequate pain control and open new avenues for personalized pain treatment.

## Supporting Information

S1 Fig Illustration of personalized medicine in patients with high and low opioid sensitivity. The minimal effective analgesic concentration (MEAC) is 5- to 10-fold different among individuals, and this is a purported cause of wide variations in the clinical response to opioids among individuals. The difference between the MEAC and maximum concentration with pain (MCP) is low among individuals. Purple, pink, blue, and brown zones indicate ranges of the blood opioid concentration associated with severe pain (no analgesia), pain (insufficient analgesia), satisfactory analgesia, and side-effects, respectively. Conventional pain control (dashed line) can result in an overdose (associated with side-effects) in patients with high opioid sensitivity or under-dose (associated with persistent pain) in patients with low opioid sensitivity. Personalized pain control (solid line) provides satisfactory pain relief in both patients.
(TIF)

S1 Table Detailed data of the subjects who underwent cosmetic orthognathic surgery. (XLSX)

S2 Table Detailed data of the subjects who underwent major open abdominal surgery. (XLSX)

S3 Table Actual fentanyl use after cosmetic orthognathic surgery stratified by genotype of the five SNPs. (DOCX)

S4 Table Actual analgesic use equivalent to systemic fentanyl after major open abdominal surgery stratified by genotype of the five SNPs. (DOCX)
