# Supplementary figures and images for: Prediction Formulas for Individual Opioid Analgesic Requirements Based on Genetic Polymorphism Analyses
Source: PLoS One. 2015 Jan 23;10(1):e0116885. doi: 10.1371/journal.pone.0116885 (PMC4304713; doi:10.1371/journal.pone.0116885)

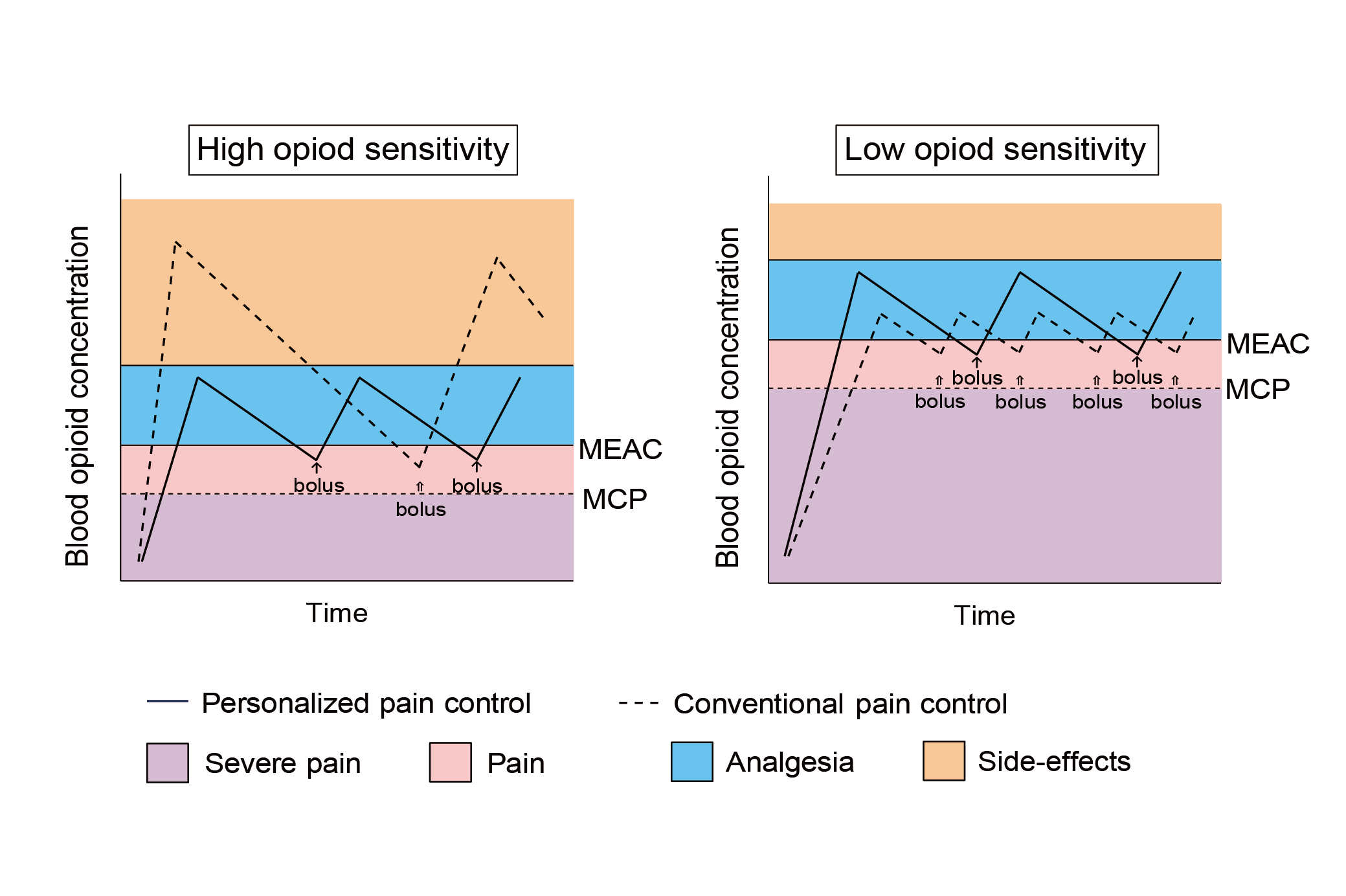

Supplement: S1 Fig — The minimal effective analgesic concentration (MEAC) is 5- to 10-fold different among individuals, and this is a purported cause of wide variations in the clinical response to opioids among individuals. The difference between the MEAC and maximum concentration with pain (MCP) is low among individuals. Purple, pink, blue, and brown zones indicate ranges of the blood opioid concentration associated with severe pain (no analgesia), pain (insufficient analgesia), satisfactory analgesia, and side-effects, respectively. Conventional pain control (dashed line) can result in an overdose (associated with side-effects) in patients with high opioid sensitivity or under-dose (associated with persistent pain) in patients with low opioid sensitivity. Personalized pain control (solid line) provides satisfactory pain relief in both patients. (TIF) [file pone.0116885.s001.tif]
