# Supplementary material for: Prediction Formulas for Individual Opioid Analgesic Requirements Based on Genetic Polymorphism Analyses
Source: PLoS One. 2015 Jan 23;10(1):e0116885. doi: 10.1371/journal.pone.0116885 (PMC4304713; doi:10.1371/journal.pone.0116885)
Supplement: S3 Table — (DOCX) [file pone.0116885.s004.docx]

**Table S3. Actual fentanyl use after cosmetic orthognathic surgery stratified by genotype of the five SNPs.**

|  | 24-h Postoperative fentanyl use (μg/kg) | Perioperative fentanyl use (μg/kg) |
| --- | --- | --- |
| *OPRM1* (rs9384179) AA/AG, GG | 1.20 ± 0.65 / 1.04 ± 0.60 | 2.20 ± 0.34 / 2.11 ± 0.34 |
| *CACNA1E* (rs3845446) AA/AG, GG | 1.26 ± 0.65 / 1.09 ± 0.62 | 2.23 ± 0.34 / 2.13 ± 0.34 |
| *ADRB2* (rs11959113) AA, AG/GG | 1.25 ± 0.58 / 1.10 ± 0.68 | 2.23 ± 0.31 / 2.14 ± 0.36 |
| *GIRK2* (rs2835859) TT/TC, CC | 1.21 ± 0.64 / 0.90 ± 0.59 | 2.20 ± 0.35 / 2.09 ± 0.31 |
| *CREB1* (rs2952768) CC/TC, TT | 1.67 ± 0.49 / 1.11 ± 0.64 | 2.40 ± 0.24 / 2.16 ± 0.34 |

The data are expressed as mean ± SD.
