# Supplementary material for: Prediction Formulas for Individual Opioid Analgesic Requirements Based on Genetic Polymorphism Analyses
Source: PLoS One. 2015 Jan 23;10(1):e0116885. doi: 10.1371/journal.pone.0116885 (PMC4304713; doi:10.1371/journal.pone.0116885)
Supplement: S4 Table — (DOCX) [file pone.0116885.s005.docx]

**Table S4. Actual analgesic use equivalent to systemic fentanyl after major open abdominal surgery stratified by genotype of the five SNPs.**

|  | 24-h Postoperative analgesic use (μg/kg) | Perioperative analgesic use (μg/kg) |
| --- | --- | --- |
| *OPRM1* (rs9384179) AA/AG, GG | 0.39 ± 0.55 / 0.37 ± 0.44 | 2.25 ± 0.39 / 2.05 ± 0.49 |
| *CACNA1E* (rs3845446) AA/AG, GG | 0.35 ± 0.52 / 0.43 ± 0.53 | 2.23 ± 0.47 / 2.19 ± 0.37 |
| *ADRB2* (rs11959113) AA, AG/GG | 0.35 ± 0.53 / 0.42 ± 0.53 | 2.22 ± 0.39 / 2.20 ± 0.44 |
| *GIRK2* (rs2835859) TT/TC, CC | 0.40 ± 0.54 / 0.30 ± 0.41 | 2.23 ± 0.42 / 2.04 ± 0.32 |
| *CREB1* (rs2952768) CC/TC, TT | 0.70 ± 0.63 / 0.35 ± 0.50 | 2.36 ± 0.44 / 2.19 ± 0.41 |

The data are expressed as mean ± SD.
